# Immunogenicity of BNT162b2 mRNA COVID-19 Vaccine in Patients with Cardiovascular Disease

**DOI:** 10.3390/jcm10235498

**Published:** 2021-11-24

**Authors:** Hiroyuki Naruse, Hiroyasu Ito, Hideo Izawa, Masayoshi Sarai, Junnichi Ishii, Eirin Sakaguchi, Reiko Murakami, Tatsuya Ando, Hidetsugu Fujigaki, Kuniaki Saito

**Affiliations:** 1 Department of Clinical Pathophysiology, Fujita Health University Graduate School of Health Sciences, Toyoake 470-1192, Japan; sakaguch@fujita-hu.ac.jp; 2 Department of Joint Research Laboratory of Clinical Medicine, Fujita Health University School of Medicine, Toyoake 470-1192, Japan; hiroyasu.ito@fujita-hu.ac.jp (H.I.); taniyama@fujita-hu.ac.jp (R.M.); andotatstuya@yahoo.co.jp (T.A.); 3 Department of Cardiology, Fujita Health University School of Medicine, Toyoake 470-1192, Japan; izawa@fujita-hu.ac.jp (H.I.); msarai@fujita-hu.ac.jp (M.S.); 4 Bantane Hospital, Fujita Health University School of Medicine, Nagoya 454-8509, Japan; jishii@fujita-hu.ac.jp; 5 Department of Advanced Diagnostic System Development, Fujita Health University Graduate School of Health Sciences, Toyoake 470-1192, Japan; fujigaki@fujita-hu.ac.jp (H.F.); saitok@fujita-hu.ac.jp (K.S.)

**Keywords:** coronavirus disease 2019 (COVID-19), vaccine, cardiovascular disease, immunogenicity

## Abstract

Concern has been raised about the effectiveness of the coronavirus disease 2019 (COVID-19) vaccine in the population of patients with various comorbidities such as heart disease. We investigated the humoral response to the BNT162b2 mRNA COVID-19 vaccine in patients with cardiovascular disease (CVD). We measured IgG against severe acute respiratory syndrome coronavirus 2 spike receptor-binding domain (RBD-IgG) in 85 CVD patients and 179 healthcare workers (HCWs). Blood samples were collected from patients and HCWs three times: (1) before the first dose of vaccination, (2) two weeks after the first dose of vaccination, and (3) two weeks after the second dose of vaccination. Patients with CVD showed a significantly inferior serological response to the BNT162b2 mRNA COVID-19 vaccine at 14 days after the prime dose compared to HCWs (21% vs. 95%, *p* < 0.001). Median RBD-IgG titers of patients with CVD at 14 days after the second dose were significantly lower than those of HCWs (137.2 U/mL (80.6–200.4 U/mL) vs. 176.2 U/mL (123.9–260.0 U/mL), *p* < 0.001). In multivariable analyses, CVD is significantly associated with seropositivity after first vaccination and RBD-IgG titers after second vaccination. CVD patients may have a poor humoral response to the BNT162b2 mRNA COVID-19 vaccine, need to be closely monitored, and require earlier revaccination to ensure stronger immunity and protection against infection.

## 1. Introduction

The coronavirus disease 2019 (COVID-19) pandemic caused by severe acute respiratory syndrome coronavirus 2 (SARS-CoV-2) has had a widespread impact on health, including a substantial mortality among patients with various pre-existing health conditions [1]. Patients with cardiovascular disease (CVD) are more susceptible to the development of severe COVID-19 infection [2]. The incidence of mechanical complications of acute coronary syndrome (ACS) increased fivefold after the declaration of a state of emergency in Japan [3]. Therefore, vaccination against SARS-CoV-2 is generally recommended in patients with CVD, as is vaccination against other infectious agents. The BNT162b2 mRNA COVID-19 vaccine has shown promising efficacy and safety, mainly in people without apparent pre-existing comorbidities [4,5]. A nationwide mass vaccination study focused on the estimated vaccine effectiveness of patients with various comorbidities such as heart disease [6]. No data, however, are available regarding the vaccine effectiveness in patients with CVD alone. This prompted us to investigate the humoral response of patients with CVD to the BNT162b2 mRNA COVID-19 vaccine compared to that in healthcare workers (HCWs).

## 2. Materials and Methods

### 2.1. Study Design and Participants

This prospective study was conducted at the Department of Clinical Pathophysiology, Fujita Health University Graduate School of Health Sciences, in collaboration with the Department of Cardiology, Fujita Health University School of Medicine (Toyoake, Japan). Between March and May 2021, outpatients with CVD who received the BNT162b2 mRNA COVID-19 vaccine (Pfizer–BioNTech), administered in 2 doses given 3 weeks apart, were enrolled. Patients who had the following characteristics were excluded from the study: (1) suspected current SARS-CoV-2 infection, such as those with fever and/or respiratory symptoms, (2) a previous diagnosis of COVID-19, (3) clinical or electrocardiographic evidence suggestive of an ACS or coronary revascularization during the previous 6 months, (4) symptomatic heart failure, (5) having an active malignant disease being treated with chemotherapy or radiation, and (6) having any autoimmune diseases. HCWs who received the BNT162b2 mRNA COVID-19 vaccine in our hospital vaccination program and without a history of COVID-19 were invited via the internet to participate in this study.

The ethics committee of Fujita Health University approved this study (study protocol number HM20−599), which was in accordance with the Declaration of Helsinki. All patients individually provided written informed consent to participate in it.

### 2.2. Blood Samples and Antibody Test

Blood samples were collected from patients and HCWs 3 times: (1) before the first dose of vaccination, (2) two weeks after the first dose of vaccination, and (3) two weeks after the second dose of vaccination. Serum was obtained by centrifugation for 15 min at 1500 g at room temperature, aliquoted and stored at −80 °C until use. Immunogenicity was measured as IgG against SARS-CoV-2 spike receptor-binding domain (RBD-IgG) using an enzyme-linked immunosorbent assay (ELISA) kit from FUJIFILM Wako Pure Chemical Corporation. All procedures were performed in accordance with the manufacturer’s instructions. RBD-IgG > 0.86 U/mL is considered a positive result as previously reported [7].

### 2.3. Definitions

Coronary artery disease was defined as a history of documented myocardial infarction, prior coronary revascularization, chest pain with documented myocardial ischemia on noninvasive tests, or coronary stenosis of >50% proven by angiography. Arrhythmia was defined as a prior catheter ablation for supraventricular or ventricular tachycardia or permanent pacemaker implantation. Hypertensive heart disease included history of hypertension and left ventricular wall thickness in the absence of other causes. Cardiomyopathy was defined as the presence of a dilated or hypertrophic cardiomyopathy. Aortic dissection or aneurysm and valvular disease were diagnosed by imaging findings such as computed tomography and transthoracic echocardiography. Hypertension was defined as having a systolic blood pressure of ≥140 mmHg, a diastolic blood pressure of ≥90 mmHg, or a history of antihypertensive treatment. Dyslipidemia was defined as a total cholesterol level of ≥220 mg/dL or a history of lipid-lowering therapy. Diabetes was defined as having a history of or current diabetes, a fasting plasma glucose level of ≥126 mg/dL, a hemoglobin A1c value of ≥6.5%, or the presence of diabetic retinopathy. Allergic disease was defined as having allergic rhinitis, hay fever, urticaria, and/or bronchial asthma.

### 2.4. Statistical Analysis

All statistical analyses were performed using StatFlex version 6 (Artech Co. Ltd., Osaka, Japan). Normally distributed variables are expressed as mean values ± standard deviations, whereas nonparametric data are presented as medians and interquartile ranges. Intergroup differences were evaluated by the chi-square test for categorical variables. A Wilcoxon signed-rank test was used to compare paired non-parametric data. The Mann–Whitney U test was used to analyze non-normally distributed data. All baseline variables with *p* < 0.05 in univariate analyses were integrated into the multivariate model to determine the independent predictors of positive serological response and RBD-IgG values. We fitted binary logistic regression models for the positive serological response including age as a continuous variable, and sex, allergic disease, hypertension, dyslipidemia, and diabetes as categorical variables. Multivariable regression analyses were performed by fitting a generalized linear model on the RBD-IgG values. A *p* value < 0.05 was considered significant.

## 3. Results

### 3.1. Baseline Characteristics of the Study Participants

The demographics and clinical characteristics of the participants are summarized in Table 1. A total of 264 participants were enrolled in this study: 85 patients with CVD (median (interquartile range) age, 74 (68–77) years; 67 men) and 179 HCWs (49 (41–55) years; 58 men). Among patients, the most common diagnosis was coronary artery disease (53 patients (63%)), followed by arrhythmia (9 patients (11%)) and hypertensive heart disease (10 patients (12%)). Intervals between the first and second dose of vaccinations and serum sampling were comparable between patients (14.7 ± 1.9, 14.9 ± 1.7 days) and HCWs (14.7 ± 1.7, 14.3 ± 1.6 days).

### 3.2. Seropositivity after Vaccination

Among all participants, 188 (71%) were seropositive for RBD-IgG (>0.86 U/mL) at 14 days after the prime dose. The prevalence of seropositivity in patients with CVD after the prime dose was significantly lower compared to that of HCWs (21% vs. 95%, *p* < 0.001). After adjusting for covariables, patients with CVD (compared to HCWs) were associated with a low positive serological response (Table 2). All participants had developed a positive antibody response by 14 days after the booster dose.

### 3.3. Antibody Titers after Vaccination

**Figure 1:**
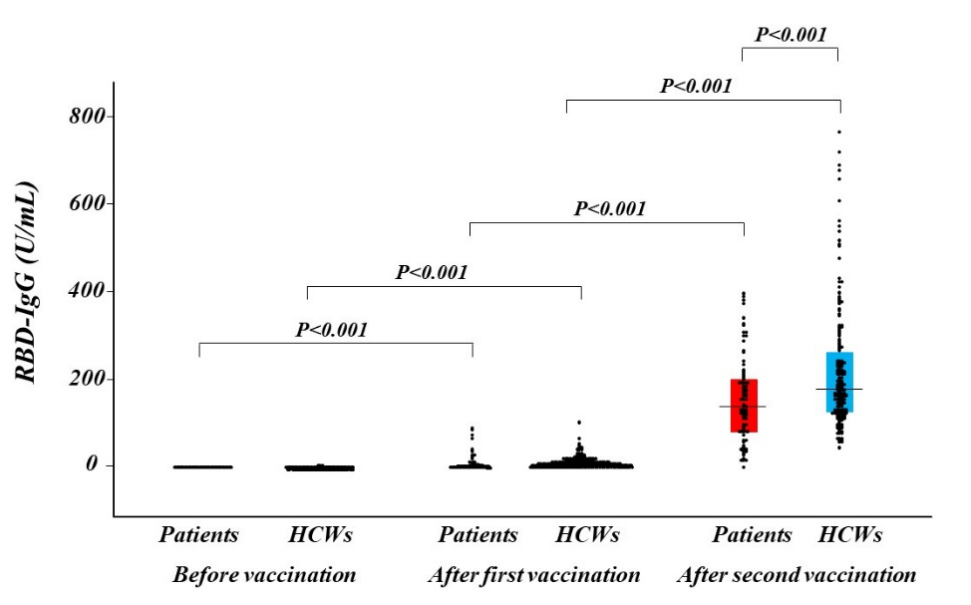
Distribution of antibody titer. Humoral quantitative IgG against SARS-CoV-2 spike RBD response at before vaccination, after the first and the second of vaccination in patients with CVD (red box) and in HCWs (blue box). The dots depict antibody levels. A box represents interquartile range and a horizontal line in a box represents the median. CVD, cardiovascular disease; HCWs, healthcare workers; RBD, receptor-binding domain; SARS-CoV-2, severe acute respiratory syndrome coronavirus 2.

RBD-IgG titers at 14 days after the prime and booster doses increased compared with pre-vaccination and dramatically increased after the booster dose in both the patient and HCW groups (Figure 1). Median RBD-IgG titers at 14 days after the booster were significantly lower in patients with CVD than in HCWs (137.2 U/mL (80.6–200.4 U/mL) vs. 176.2 U/mL (123.9–260.0 U/mL), *p* < 0.001). In a multivariable regression analysis, there was a significant association between patients with CVD (compared to HCWs) and low RBD-IgG titers after the booster dose (Table 3).

## 4. Discussion

The main findings of this prospective study were as follows. First, patients with CVD showed a significantly inferior serological response to the BNT162b2 mRNA COVID-19 vaccine at 14 days after the prime dose compared to HCWs. Second, median RBD-IgG titers of patients with CVD at 14 days after the booster dose were significantly lower than those of HCWs. Third and finally, CVD remained an independent predictor of lower RBD-IgG levels after adjusting for comorbidities. These findings suggest that patients with CVD may have a poor humoral response to the BNT162b2 mRNA COVID-19 vaccine, need to be closely monitored, and require earlier revaccination to ensure stronger immunity and protection against infection.

Impaired immunogenicity of the BNT162b2 mRNA COVID-19 vaccine has been reported in immunocompromised patients treated for solid tumors [8] and with solid organ transplantation [9,10,11]. However, the efficacy of the BNT162b2 mRNA COVID-19 vaccine in patients with heart disease has never been investigated. To the best of our knowledge, this is the first study to document a blunt response after a complete cycle of the BNT162b2 mRNA COVID-19 vaccine in patients with CVD.

COVID-19 vaccines demonstrate excellent efficacy in clinical trials [4,5] and effectiveness in real-world data [6], but some individuals still become infected with SARS-CoV-2 after vaccination. A recent study demonstrated that in older adults (≥60 years) who had received their first vaccine dose but not their second, heart disease was associated with post-vaccination infection [12]. Our data showed an inferior serological response to the COVID-19 vaccine after the first vaccine dose, which may be associated with susceptibility to COVID-19 infection after vaccination in patients with heart disease. Therefore, these patients may be highly recommended to receive the complete cycle of the COVID-19 vaccine. Hall et al. reported an increment in the immunogenicity of the COVID-19 vaccine with administration of a third dose in transplant recipients [13]. Our results demonstrated that patients with CVD had lower antibody levels after a booster dose compared to HCWs, suggesting that these patients should be prioritized to receive the third dose of vaccination.

In this study, differences in age and gender were observed between CVD patients and HCWs. Older age has been repeatedly reported to associate with reduced antibody responses after COVID-19 vaccination [14,15,16]. Female gender has also been shown to correlate with antibody responses to COVID-19 vaccination to various degrees [17]. These differences may make it difficult to evaluate the humoral response to COVID-19 vaccination in patients with CVD. In multivariate analyses, however, CVD was independently associated with seropositivity after the first vaccination and RBD-IgG titers after the second vaccination. Furthermore, when we performed multivariate analyses in a subgroup of 16 HCWs over 60 years (median age 65 years; male, 63%) and patients with CVD, similar results were obtained (data not shown). Consequently, we believe that these factors did not significantly affect our results. The mechanisms that emphasize the association between CVD and a poor humoral response to the BNT162b2 mRNA COVID-19 vaccine are still unclear. It is possible that the medications used may be associated with poorer humoral response in patients with CVD. In the present study, there were no significant differences in humoral response according to their medications, such as renin–angiotensin–aldosterone system inhibitors, beta-blockers, and statins. Further investigations are required to clarify this issue.

To date, some COVID-19 vaccines have been approved for emergency use. Diverse platforms have been used to deliver the recombinant SARS-CoV-2 spike, such as mRNA-encapsulating liposomes, adenovirus vectors, and micelle-attached spikes [18]. The humoral response may be different according to the type of vaccine. A notable loss of vaccine efficacy against SARS-CoV-2 variants is reported, likely caused by spike mutations in the RBD, N-terminal domain, and other regions [19,20]. It may be necessary to investigate the neutralizing antibody against variants of SARS-CoV-2.

Study limitations include a small sample size, single-center study, and a paucity of long-term data. Antibody response against vaccines does not give objective information about immunogenicity and immunoprotection. Thieme et al. showed that mounted cellular response could be sufficient to protect against COVID-19, despite lower humoral immunity in hemodialysis patients after BNT162b2 mRNA COVID-19 vaccination [21]. Oberhardt et al. reported that spike-specific CD8+ T cell response is elicited already after prime vaccination at a time point when neutralizing antibodies were hardly detectable and coincided with the protective effect [22]. We did not evaluate T-cell and B-cell-mediated responses, which could counterbalance the impaired humoral response. Therefore, we should pay attention to the clinical interpretation of our results. Deeper immunophenotyping of patients with CVD after vaccination will be important in deciding future vaccination strategies. In spite of growing insights into the persistence and decline of antibody responses following vaccination [23,24], we do not yet know the exact correlates of immunity regarding the levels of required antibody titers. Further studies will be needed to clarify how lower antibody titers might affect COVID-19 morbidity.

## 5. Conclusions

Patients with CVD may have a poor humoral response to the BNT162b2 mRNA COVID-19 vaccine, need to be closely monitored, and require earlier revaccination to ensure stronger immunity and protection against infection.

## Figures and Tables

**Table 1:** Baseline characteristics of the study participants.

	Patients (*n* = 85)	HCWs (*n* = 179)
Age, y	74 (68–77)	49 (41–55)
Male	67 (79)	58 (32)
Hypertension	56 (66)	16 (9)
Dyslipidemia	58 (68)	5 (3)
Diabetes	26 (31)	1 (1)
Allergic disease	8 (9)	86 (48)
Diagnosis		
Coronary artery disease	53 (63)	NA
Arrhythmia	9 (11)	
Hypertensive heart disease	10 (12)	
Cardiomyopathy	6 (7)	
Aortic dissection or aneurysm	4 (5)	
Valvular disease	3 (4)	
Previous myocardial infarction	26 (31)	
Previous coronary revascularization	38 (45)	
Paroxysmal or persistent AF	12 (14)	
Medications		
RAAS inhibitors	41 (48)	NA
Beta-blockers	34 (40)	
Diuretics	8 (9)	
Statins	46 (54)	
Antiplatelet drugs	37 (44)	
Anticoagulant drugs	16 (19)	
Intervals between the first vaccination and sampling, day	14.7 ± 1.9	14.7 ± 1.7
Intervals between the second vaccination and sampling, day	14.9 ± 1.7	14.3 ± 1.6

Data are expressed as median (25th–75th percentile), number (%), or mean ± standard deviation. NA, not applicable. HCWs, healthcare workers; AF, atrial fibrillation; RAAS, renin–angiotensin–aldosterone system.

**Table 2:** Multivariable logistic analysis of seropositive status after first vaccination.

Variables	Univariable Analysis			Multivariable Analysis		
	Odds Ratio	95% CI	*p* Value	Odds Ratio	95% CI	*p* Value
Patients with CVD (vs. HCWs)	0.01	0.01 to 0.03	<0.001	0.08	0.02 to 0.33	<0.001
Age (per 1 year increment)	0.86	0.82 to 0.89	<0.001	0.95	0.90 to 0.99	0.02
Male	0.19	0.10 to 0.34	<0.001	0.78	0.30 to 2.00	0.61
Allergic disease	5.33	2.58 to 11.0	<0.001	1.16	0.39 to 3.45	0.79
Hypertension	0.09	0.05 to 0.16	<0.001	0.60	0.21 to 1.74	0.35
Dyslipidemia	0.05	0.03 to 0.10	<0.001	0.88	0.26 to 2.95	0.84
Diabetes	0.07	0.02 to 0.19	<0.001	0.63	0.19 to 2.10	0.45

Multivariate model adjusted for all baseline variables with *p* < 0.05 by univariate analysis. CI, confidence interval; CVD, cardiovascular disease; HCWs, healthcare workers.

**Table 3:** Multivariable regression analysis of RBD-IgG titers after a complete cycle of vaccination.

Variables	Univariable Analysis			Multivariable Analysis		
	β Coefficient	95% CI	*p* Value	β Coefficient	95% CI	*p* Value
Patients with CVD (vs. HCWs)	−0.22	−0.34 to −0.10	<0.001	−0.32	−0.60 to −0.04	0.02
Age (per 1 year increment)	−0.14	−0.27 to −0.02	0.03	0.12	−0.08 to 0.31	0.25
Male	−0.22	−0.34 to −0.10	<0.001	−0.17	−0.30 to −0.03	0.02
Hypertension	−0.21	−0.33 to −0.08	0.001	−0.17	−0.34 to 0.01	0.06
Dyslipidemia	−0.14	−0.26 to −0.01	0.03	0.17	−0.03 to 0.38	0.10
Diabetes	−0.14	−0.26 to −0.01	0.03	−0.04	−0.18 to 0.10	0.60

Multivariate model adjusted for all baseline variables with *p* < 0.05 by univariate analysis. CI, confidence interval; CVD, cardiovascular disease; HCWs, healthcare workers.

## Data Availability

The data presented in this study are available from the corresponding author on reasonable request.
